# Heart Regeneration in the Mexican Cavefish

**DOI:** 10.1016/j.celrep.2018.10.072

**Published:** 2018-11-20

**Authors:** William T. Stockdale, Madeleine E. Lemieux, Abigail C. Killen, Juanjuan Zhao, Zhilian Hu, Joey Riepsaame, Noémie Hamilton, Tetsuhiro Kudoh, Paul R. Riley, Ronny van Aerle, Yoshiyuki Yamamoto, Mathilda T.M. Mommersteeg

**Affiliations:** 1 Department of Physiology, Anatomy and Genetics, University of Oxford, South Parks Road, Oxford, OX1 3PT, UK; 2 Bioinfo, Plantagenet, ON K0B 1L0, Canada; 3 Genome Engineering Oxford, Sir William Dunn School of Pathology, University of Oxford, South Parks Road, Oxford, OX1 3RE, UK; 4 Department of Cell and Developmental Biology, University College London, London, WC1E 6BT, UK; 5 Biosciences, College of Life and Environmental Sciences, Geoffrey Pope Building, University of Exeter, Stocker Road, Exeter, EX4 4QD, UK; 6 Centre for Environment, Fisheries and Aquaculture Science (Cefas), Weymouth, Dorset DT4 8UB, UK

**Keywords:** Mexican cavefish, heart regeneration, fibrotic scar, QTL, myocardial proliferation, *lrrc10*

## Abstract

Although *Astyanax mexicanus* surface fish regenerate their hearts after injury, their Pachón cave-dwelling counterparts cannot and, instead, form a permanent fibrotic scar, similar to the human heart. Myocardial proliferation peaks at similar levels in both surface fish and Pachón 1 week after injury. However, in Pachón, this peak coincides with a strong scarring and immune response, and ultimately, cavefish cardiomyocytes fail to replace the scar. We identified *lrrc10* to be upregulated in surface fish compared with Pachón after injury. Similar to cavefish, knockout of *lrrc10* in zebrafish impairs heart regeneration without affecting wound cardiomyocyte proliferation. Furthermore, using quantitative trait locus (QTL) analysis, we have linked the degree of heart regeneration to three loci in the genome, identifying candidate genes fundamental to the difference between scarring and regeneration. Our study provides evidence that successful heart regeneration entails a delicate interplay between cardiomyocyte proliferation and scarring.

## Highlights

• *Astyanax mexicanus* surface fish regenerate their hearts, but Pachón cavefish cannot
• Successful regeneration is a delicate interplay between proliferation and scarring
• *lrrc10* is required for heart regeneration
• Using QTL analysis, we have identified three genomic loci linked to heart regeneration

## Graphical Abstract

**Figure:**
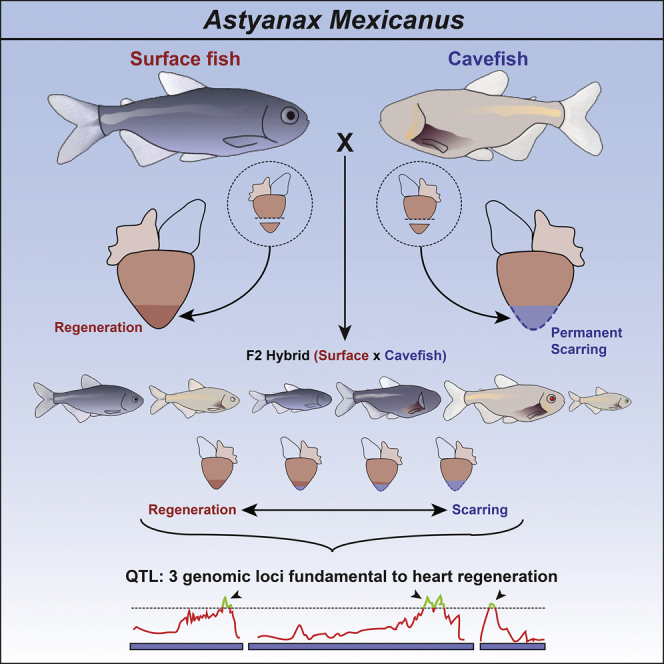

## Teaser

Stockdale et al. identify the Mexican cavefish as a model for heart regeneration research. This model provides an opportunity for direct comparison of natural regeneration versus scarring within a single species. Furthermore, they have identified key genomic regions regulating heart regeneration using an unbiased quantitative trait loci analysis approach.

## Introduction

**Figure 1:**
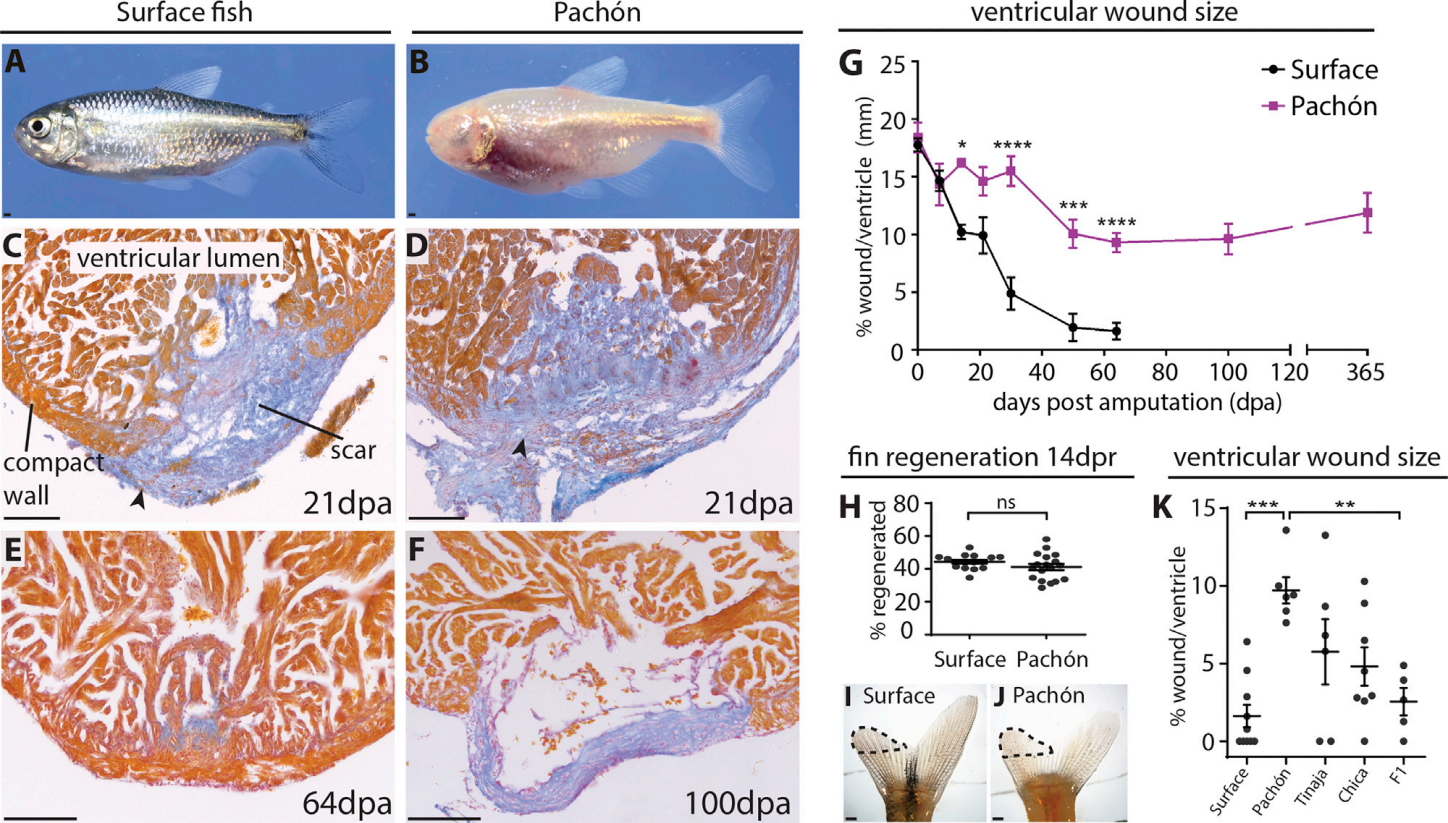
Permanent Scar Formation after Ventricular Resection in the Pachón Cavefish (A and B) Adult surface (A) and Pachón cavefish (B). (C–F) AFOG staining of the ventricular apex after resection. AFOG staining is a technique that stains myocardium orange and collagen blue. Both populations form a collagen scar (C and D, arrowheads), which disappears in the surface fish around 64 dpa (E), but persists in the Pachón (F). (G) Time line showing the reducing wound size in the surface fish but persisting wound in the Pachón. n ≥ 3 per population per time point, two-way ANOVA with Sidak’s test. (H) No difference in fin regeneration between Pachón (n = 18) and surface fish (n = 16) at 14 days post resection (dpr). Unpaired t test. (I and J) Regenerating dorsal lobes of tail fins of surface fish (I) and Pachón (J) at 14 dpr. Dotted line indicates the regenerated part. (K) Wound size 64 dpa in surface fish (n = 10), Pachón (n = 7), Tinaja (n = 6), Chica (n = 8), and surface fish × Pachón F1 hybrids (n = 5). One-way ANOVA with Tukey’s test. Detailed numbers and statistics are reported in STAR Methods Results are presented as mean ± SEM. All scale bars, 100 μm.

Complete regeneration of the adult heart after injury is a feature exclusive to a limited number of species, including vertebrates such as the zebrafish and salamander (Borchardt and Braun, 2007, Poss et al., 2002, Witman et al., 2011). Injury to a zebrafish heart results in a scar-free regeneration process, with the wound tissue completely being replaced by new, functional cardiac muscle (Chablais et al., 2011, González-Rosa et al., 2011, Kikuchi et al., 2010, Poss et al., 2002). In contrast, in patients fortunate enough to survive a heart attack (myocardial infarction), the dead heart muscle is replaced by a permanent scar that may cause severe contractile dysfunction, resulting in heart failure and even recurring myocardial infarction (Gerbin and Murry, 2015). Identification of the fundamental mechanisms driving natural heart regeneration in fish could lead to the development of strategies to heal the human heart after injury. However, to date, research on fish heart regeneration has not yet led to significant breakthroughs in the search for therapies achieving human heart regeneration. This is largely because the use of candidate gene approaches is biased toward the identification of genes and molecular mechanisms already predicted to affect the ability of the heart to regenerate. Second, even though the zebrafish has proved to be a useful model to study human diseases, it is difficult to identify novel genes purely regulating heart regeneration versus scarring while directly comparing different species with divergent physiologies. Comparing the natural regenerative and scarring response within the same species would avoid confounding physiological factors and allow identifying the key mechanisms driving regeneration, aiding the translation of advances in our understanding of fish heart regeneration to human. Here, we present a fish model for heart regeneration research that overcomes these problems, *Astyanax mexicanus*. *Astyanax mexicanus*, a teleost fish like the zebrafish, is a single fish species comprising cave-dwelling and surface populations (Figures 1A and 1B), both of which are easily maintained in the laboratory. About 1.5 million years ago, surface fish, living in rivers in northern Mexico, diverged into at least 29 distinct caves. Although some caves are interconnected, there are at least four verified independent cave populations (Gross, 2012). During their independent evolution in caves, the fish lost certain features, such as their eyes and pigment, redundant traits in the absence of light, while gaining other characteristics, for example, developing highly sensitive taste buds and lateral line systems specialized for finding food in the dark (Jeffery, 2009). In addition, the fish changed their metabolism to allow for long periods of food scarcity in the caves (Aspiras et al., 2015, Riddle et al., 2018). The value of *Astyanax mexicanus* as a research model lies in the ability to compare these different traits within the same species. Furthermore, the ability to interbreed between the surface fish and cavefish allows genetic mapping approaches to identify loci associated with specific phenotypic changes (Protas et al., 2006). Here, we show that *Astyanax mexicanus* cavefish and surface fish respond differently to cardiac injury. This not only allows us to compare the regenerative and scarring response within one species but also provides the opportunity to link the capacity for heart regeneration directly to the genome using forward genetic screening.

## Results

### Pachón Cavefish do Not Regenerate Their Hearts after Injury

After surgical removal of the ventricular apex, surface fish (Figure 1A) were able to regenerate their hearts completely, while cavefish from the Pachón cave (Figure 1B) could not and instead formed a permanent fibrotic scar, similar to the human injury response (Figures 1C–1G). In contrast to zebrafish, in which the resection wound largely consists of fibrin (Poss et al., 2002), both Pachón and regenerating surface fish hearts laid down an initial extensive collagen scar (Figures 1C and 1D, arrowheads). This likely explains the increased time required to complete myocardial regeneration compared with the 30–60 days after resection in zebrafish (Figure 1G) (Poss et al., 2002) and is more similar to the collagen scar that results from cryo-injury in zebrafish (Chablais et al., 2011, González-Rosa et al., 2011). Whereas 50% of surface fish hearts had regenerated their myocardium 64 days post amputation (dpa) (Figures 1G and 1K), all Pachón cavefish, even if examined after 1 year, retained large collagen scars (Figure 1G). Although the decrease in wound size in Pachón between 30 and 64 dpa (Figure 1G) could suggest partial regeneration, it coincided with a period of extensive remodeling of the large initial collagen network into a much thinner scar (Figures 1D and 1F). The absence of regeneration in Pachón seems specific to the heart, as fin regeneration was not significantly impaired in these fish (Figures 1H–1J). The complete block on heart regeneration also appears to be specific to fish from the Pachón cave. Heart regeneration in fish from both the Tinaja and Chica caves showed an interesting range from not regenerating to completely regenerated at 64 dpa (Figure 1K).

### Similar Levels of Cardiomyocyte Proliferation between Surface Fish and Pachón after Injury

**Figure 2:**
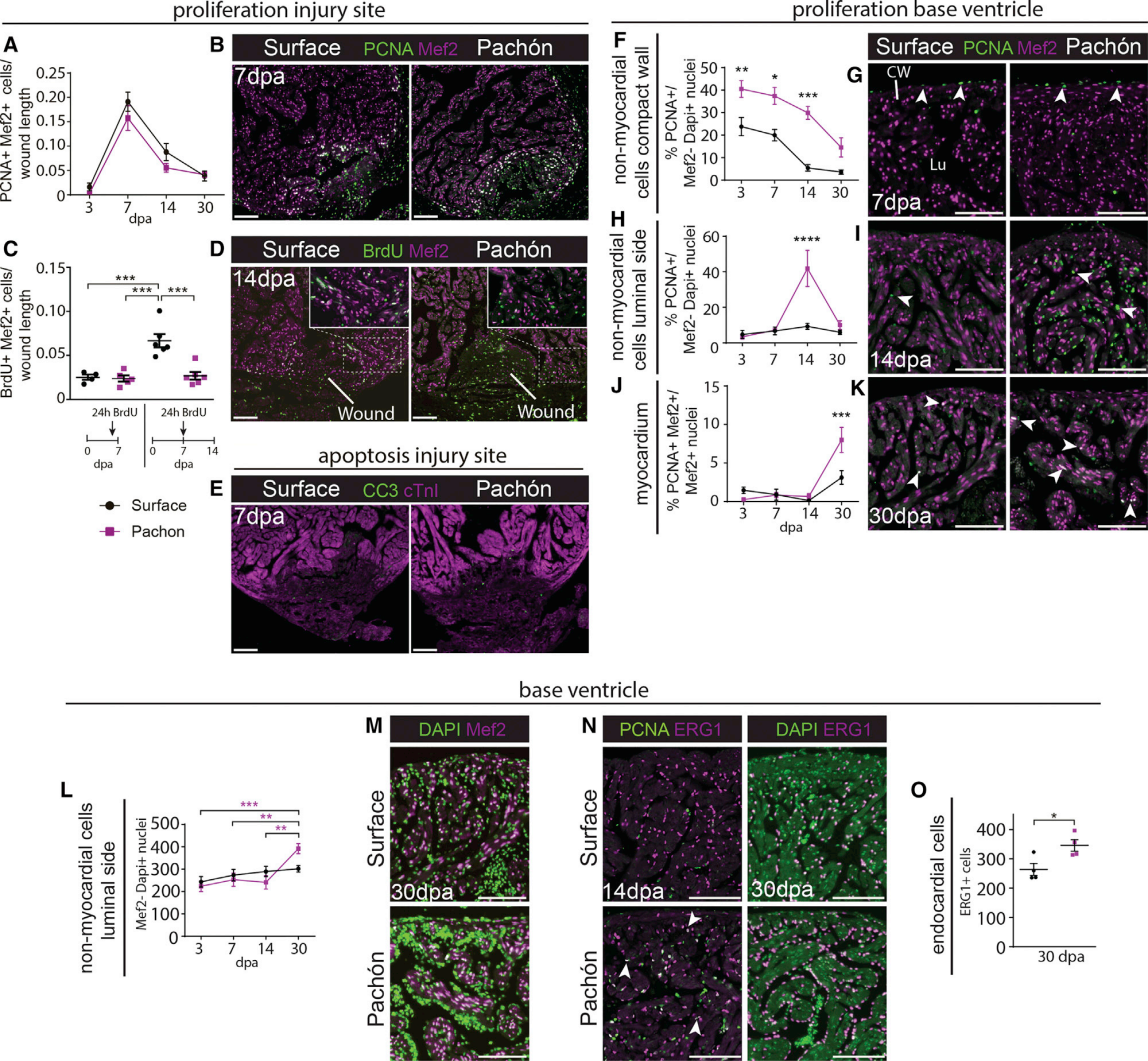
Myocardial Proliferation but No Regeneration in Pachón Hearts (A and B) No significant difference in the number of PCNA/Mef2-positive cells surrounding the wound in Pachón compared with surface fish hearts (A). Myocardial proliferation is highest at 7 dpa in both fish (B). n ≥ 4 per population per time point, two-way ANOVA with Sidak or Tukey test. (C and D) Twenty-four-hour BrdU administration at 6 dpa with heart isolation at 7 dpa or 24 hr BrdU at 7 dpa with isolation at 14 dpa (C). No difference in the number of BrdU-positive cells at 7 dpa (Pachón n = 5, surface fish n = 4) but reduced labeling in the Pachón (n = 6) compared with surface fish (n = 6) at 14 dpa (D). Similar number of cells labeled in Pachón at 7 and 14 dpa. One-way ANOVA with Tukey’s test. (E) Comparable low levels of CC3-positive cells in surface fish and Pachón. cTnI, cardiac troponin I. (F–K) PCNA counts on the basal side of the ventricle. n ≥ 4 per population per time point, two-way ANOVA with Sidak or Tukey test. Increased non-myocardial proliferation at the epicardial layer after injury in the Pachón compared to surface fish (F), and representative image of non-myocardial proliferation 7dpa, indicated by PCNA-positive/Mef2-negative cells at the epicardial layer (G). Sharp increase in non-myocardial proliferation at 14 dpa in the luminal cells of the ventricle in the Pachón versus surface fish (H), with representative image of non-myocardial proliferation 14 dpa in the trabecular area/luminal side (I). Increased myocardial proliferation on the other side of the ventricle in Pachón compared to surface fish at 30 dpa (J), with representative image of myocardial proliferation basal side indicated by PCNA/Mef2-positive cells at 30 dpa in surface fish and Pachón (K). (L and M) Increased non-myocardial cells in the Pachón heart at 30 dpa (L). Representative DAPI and mef2 staining in Pachón and surface fish at the base of the ventricle, 30 dpa (M). n = 4 per fish per time point, two-way ANOVA with Sidak or Tukey test. (N and O) Proliferation in the non-myocardial luminal cells is mostly located in Erg1-positive endocardial cells (N), followed by an increase in endocardial cells in Pachón at 30 dpa (O). Unpaired, two-tailed, equal-variance t test, p = 0.0274. Detailed numbers and statistics are provided in STAR Methods. Results are presented as mean ± SEM. All scale bars, 100 μm. CW, compact wall; Lu, lumen.

During zebrafish heart regeneration, a strong myocardial proliferative response is observed directly adjacent to the wound area (Jopling et al., 2010, Kikuchi et al., 2010). Interestingly, this was recapitulated in both the regenerating surface and non-regenerating Pachón hearts (Figures 2A and 2B). As observed by overlapping expression of proliferating cell nuclear antigen (PCNA) and Mef2, myocardial proliferation rates at the wound border were highest at 7 dpa in both fish (Figure 2A). These data were confirmed by exposing the fish for 24 hr to bromodeoxyuridine (BrdU) directly before isolating the hearts at 7 dpa, which again showed similar myocardial proliferation levels between the two fish (Figure 2C). To determine contribution of these proliferating cardiomyocytes to myocardial regeneration, we next exposed the fish for 24 hr to BrdU at 7 dpa, before isolating the hearts at 14 dpa. This BrdU pulse-chase revealed that although gross proliferation rates were similar within the myocardium at 7 dpa, the number of BrdU-positive cardiomyocytes contributing to the ventricle was higher in surface fish compared with Pachón (Figures 2C and 2D). There was no clear increase in myocardial apoptosis in Pachón that might have explained the apparent lack of contribution of new cardiomyocytes to the Pachón ventricle (Figure 2E). This suggests that the cells either die, through a caspase-independent pathway, re-differentiate into a non-myocardial cell type, or have defective cytokinesis.

While analyzing wound proliferation, we noticed that further away from the wound, PCNA proliferation rates did appear to be different between surface and cavefish. Analysis of these rates showed that Mef2-negative non-myocardial cells surrounding the compact wall of the ventricle proliferated at a much higher rate in Pachón after injury (Figures 2F and 2G). Fourteen days after wounding, when cardiomyocyte proliferation was reduced at the border zone, there was a rapid and significant proliferative response of non-myocardial cells throughout the trabecular area of the Pachón heart (Figures 2H and 2I), followed by an increase in non-myocardial cell numbers as well as myocardial proliferation at 30 dpa (Figures 2J–2M). Co-labeling of PCNA with endothelial cell marker ERG1 showed that in particular the endocardium is highly proliferative in Pachón at 14 dpa, resulting in an increase in the endocardial cell population at 30 dpa (Figures 2N and 2O). This suggests a more marked increase in epicardial proliferation as well as a late response in proliferation of the endocardium in Pachón that may be linked to the increased and persistent scarring.

### Upregulation of Immune and Scarring Responses in Pachón Compared with Surface Fish

**Figure 3:**
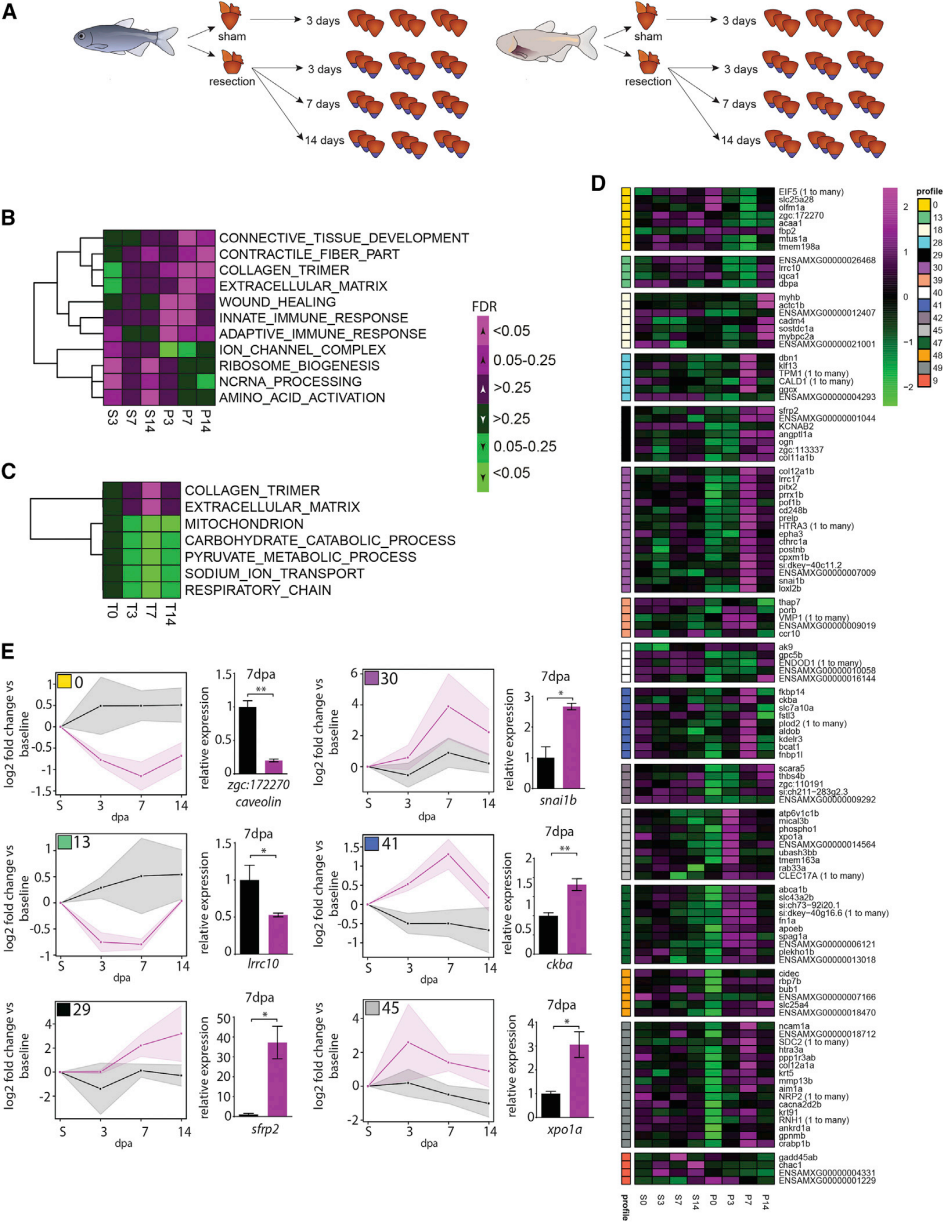
Analysis of the Kinetics of Differential Expression in Response to Wounding Identifying *lrrc10* (A) Experimental design. Three ventricles pooled per sample, three samples per time point. (B) Heatmap of signed false discovery rate (FDR) values for selected Gene Ontology (GO) terms significantly different by pre-ranked gene set enrichment analysis (GSEA; FDR < 0.05) between sham and at least at one time point per fish. (C) Heatmap of signed FDR values for selected GO terms significantly different between Pachón and surface fish per time point (pre-ranked GSEA FDR < 0.05 at least at one time point). (D) Heatmap of row-normalized gene expression with differential kinetics over the different time points grouped by profile. Profiles shown only if containing at least four genes. (E) Graphs showing median differences in kinetics of selected profiles (profile numbers and colors based on C) between surface fish (gray) and Pachón (magenta), as well as qPCR validation of one of the genes in the same profiles at 7 dpa. Shaded areas show median absolute deviation. For qPCRs, n = 3 for both Pachón and surface fish, unpaired two-tailed t test. Detailed statistics are provided in STAR Methods. Results are presented as mean ± SEM.

The lack of significant differences in myocardial proliferation was confirmed by RNA sequencing on surface fish and Pachón ventricles at 3, 7, and 14 days after resection as well as a control sham-operated group (Figure 3A). Instead, we found a strong upregulation of both immune and scarring responses in Pachón compared with surface fish (Figures 3B, 3C, and S1). Interestingly, despite adaptation to nutrient-poor conditions in the cave leading to altered metabolism of Pachón (Aspiras et al., 2015, Riddle et al., 2018), we did not observe significant differences in metabolic gene activation between the control sham hearts. However, after injury, Pachón showed a strong downregulation of both mitochondrial and glycolytic pathways compared with surface fish (Figure 3C). Differences in metabolism between fish and human, and more specifically the capacity for anaerobic glycolysis, have been proposed to correlate with the ability for heart regeneration in fish (Puente et al., 2014).

Our RNA sequencing dataset allowed us to identify molecular responses specific for the difference between heart regeneration and scarring. We concentrated on 141 genes that exhibited significant differences in kinetics over all time points (Figure S2). These genes were clustered together into profiles with similar kinetics (Figure 3D; only profiles containing at least four genes are shown). The majority of genes were upregulated in Pachón, with the surface fish response to injury being more dampened. A number of genes in different clusters were then validated by qPCR. Specifically upregulated in surface fish are *caveolin* and *lrrc10* (leucine-rich repeat containing 10) (Figures 3D and 3E). Genes specifically upregulated in Pachón include *snai1b*, important for epithelial-to-mesenchymal transition (Barrallo-Gimeno and Nieto, 2005), and *sfrp2* (secreted frizzled-related protein 2), *ckba* (creatine kinase, brain a), and *xpo1a* (exportin 1a) but also a large number of uncharacterized proteins, candidates for further study including cohorts of genes involved in extracellular matrix synthesis and turnover, and inflammation (Figures 3D and 3E).

### Mutants for *lrrc10* Show Impaired Heart Regeneration but Normal Myocardial Proliferation

**Figure 4:**
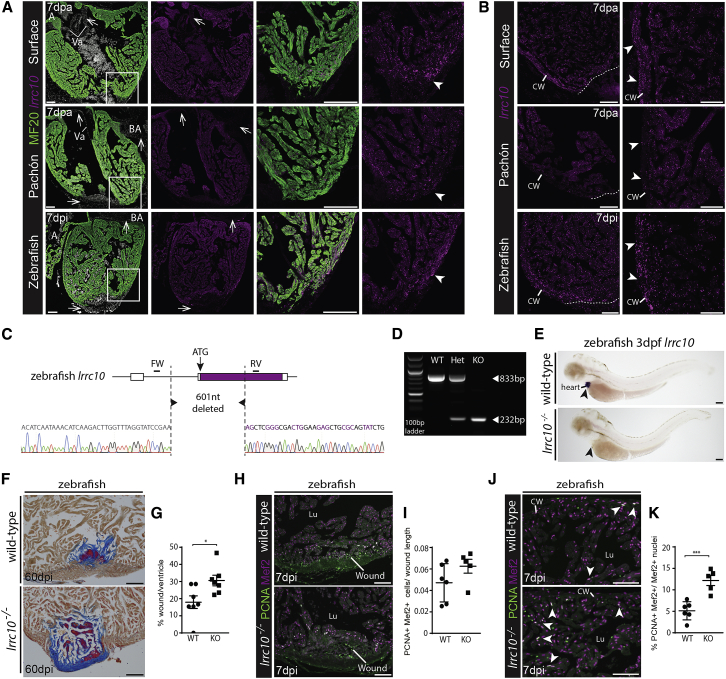
*lrrc10* Is Required for Heart Regeneration in Zebrafish (A) RNAScope RNA expression analysis shows that *lrrc10* is expressed specifically in all the MF20-positive myocardium at 7 dpa in surface fish and Pachón as well as in zebrafish at 7dpi. Arrows point to absent expression in non-myocardial tissues, such as the wound, valves, and bulbus arteriosus. Expression around the wound is higher in surface fish and zebrafish compared with Pachón (arrowheads). (B) *lrrc10* is especially higher expressed in the compact myocardium (arrowheads) compared with the trabecular myocardium close to the wound (demarcated with line) in surface fish and zebrafish. This difference is absent in Pachón. (C) Zebrafish *lrrc10* mutants were generated by removing 601 nucleotides, including the ATG start site using CRISPR/Cas9. (D) Genotyping with primers just outside the deleted region amplifies a product of 833 bp in wild-types and a product of 232 bp in mutants. (E) Whole-mount *in situ* hybridization using an *lrrc10* antisense probe in 3 dpf (days post fertilization) *lrrc10*^*−/−*^ (n = 12) and wild-type embryos (n = 25) shows that *lrrc10* expression is specifically present in the wild-type heart (arrow) but absent in the mutant, confirming knockout of *lrrc10*. (F) AFOG staining showing large blue collagen scar in the *lrrc10* mutant compared with the wild-type control at 60 dpi. (G) Wound size at 60 dpi in *lrrc10*^*−/−*^ (n = 7) and wild-type controls (n = 7), unpaired two-tailed t test. (H) PCNA/Mef2 staining on *lrrc10*^*−/−*^ and controls at 7dpi. (I) No significant difference in the number of PCNA/Mef2-positive cells surrounding the wound in *lrrc10*^*−/−*^ (n = 5) compared with wild-type hearts (n = 6) at 7 dpi. Unpaired two-tailed t test, equal variance. (J) PCNA/Mef2 staining on *lrrc10*^*−/−*^ and controls at 7dpi at the base of the ventricle. (K) Increased number of PCNA/Mef2-positive cells on the other side of the ventricle in *lrrc10*^*−/−*^ (n = 5) compared with wild-type hearts (n = 6) at 7 dpi. Unpaired two-tailed t test, equal variance. All scale bars, 100 μm. BA, bulbus arteriosus; CW, compact wall; Lu, lumen; Va, valves. Results are presented as mean ± SEM.

As *caveolin* has already been linked to heart regeneration in zebrafish (Cao et al., 2016), we tested the role of *lrrc10* during heart regeneration. We selected *lrrc10* because it is a highly conserved heart muscle-specific gene (Adameyko et al., 2005, Kim et al., 2007a, Kim et al., 2007b), associated with dilated cardiomyopathy in mouse and human (Brody and Lee, 2016, Qu et al., 2015). The fact that the gene is uniquely expressed in the heart but still has a largely unknown function makes it an interesting candidate gene to regulate heart regeneration. *lrrc10* clusters together in a profile with *iqca1*, *dbpa*, and novel gene *ENSAMXG00000026468*, which belongs to the connexin family (Figure 3D). Although these genes show similar kinetics, no interaction with *lrrc10* has been reported. *lrrc10* was specifically expressed in all the myocardium in both surface fish and Pachón at 7 dpa (Figure 4A). However, in surface fish, the areas of high myocardial proliferation (Figure 2B), close to the wound border and in particular the compact wall, showed higher expression levels of *lrrc10* compared with the rest of the heart, a difference that was not visible in Pachón (Figures 4A and 4B). As *Astyanax mexicanus* only become sexually mature when they are 1 year of age, we tested the role of *lrrc10* in zebrafish instead, for which we used the cryo-injury model to induce collagen scar formation (González-Rosa et al., 2011). *lrrc10* expression at 7 days post cryo-injury (dpi) in zebrafish was comparable with that in surface fish, with upregulation in the compact wall close to the wound (Figures 4A and 4B). Next, we generated a knockout of *lrrc10* using CRISPR-Cas9 (Figures 4C–4E), which revealed reduced regeneration 60 days after cryo-injury (Figures 4F and 4G), implicating a role for *lrrc10* during heart regeneration. Interestingly, despite the difference in regeneration, there were no observable changes in cardiomyocyte proliferation near the wound border at 7 days after injury between the wild-type fish and the *lrrc10* mutants (Figures 4H and 4I). These data confirm the validity of the RNA sequencing (RNA-seq) results, identify *lrrc10* as a factor required for zebrafish heart regeneration, and, importantly, corroborate the findings from cavefish that the ability to initiate myocardial proliferation per se is not sufficient for heart regeneration. Although proliferation at the wound border was equivalent between the *lrrc10*-mutant and control zebrafish, we observed increased proliferation throughout the rest of the ventricle in the mutants at 7 days post injury (Figures 4J and 4K). This suggests that the global cardiomyocyte proliferation observed in zebrafish after injury is also not directly linked to heart regeneration (Jopling et al., 2010).

### QTL Analysis Identifies Three Loci Linking to Heart Regeneration

**Figure 5:**
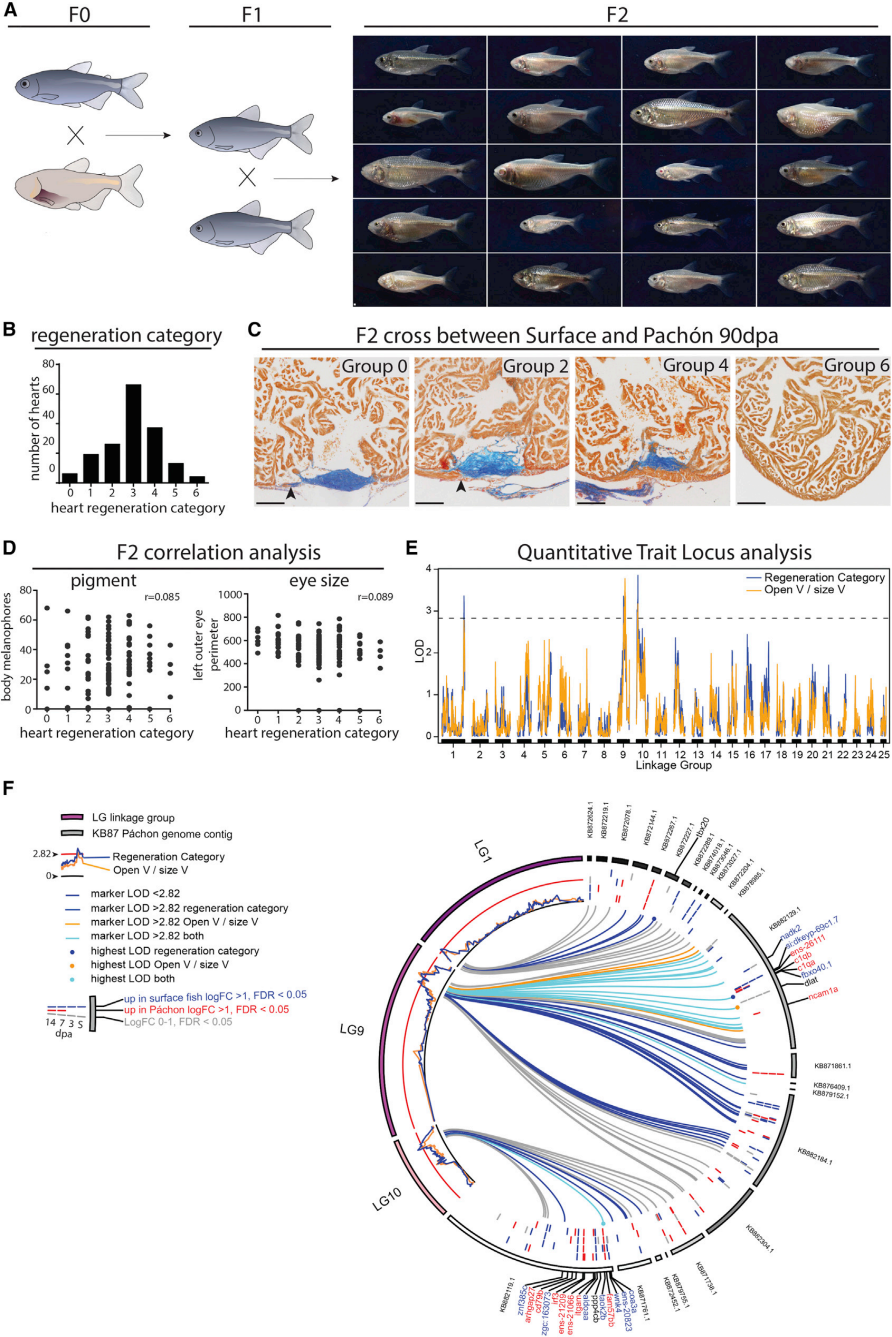
Three Loci Associated with Heart Regeneration Identified by QTL Analysis (A) The second generation of a cross between surface fish and Pachón results in offspring ranging from large, pigmented fish with no eyes to small cavefish-like fish with black eyes. One hundred eighty-eight F2 hearts were isolated 90 days after resection. (B) Number of F2 generation hearts per heart regeneration class. (C) AFOG staining on F2 hearts. Examples of four of the seven groups used for correlation tests and QTL analysis. Group 0, clear scar, neither compact wall myocardial thickening nor overgrowth (arrowhead). Group 2, clear scar, compact myocardium has started to grow over the scar (arrowhead). Group 4, clear scar, compact myocardium closed. Group 6, no sign of injury left. (D) No correlation between body melanocyte number (r = 0.085) or eye size (r = 0.089) and heart regeneration. Pearson’s correlation. (E) LOD scores per LG, showing three loci above the genome-wide significance threshold of 2.82 (95th percentile of maximum LOD from permutation analysis). (F) Circos plot mapping markers within LOD score peaks on LG1, LG9, and LG10 to the cavefish genome contigs and showing significant log_2_ fold expression changes for genes overlapping those loci. Detailed numbers and statistics are provided in STAR Methods. All scale bars, 100 μm.

A further major advantage of *Astyanax mexicanus* as a research model over zebrafish lies in the ability to inter-cross surface fish and cavefish variants and, therefore, the possibility of genetic mapping to identify loci associated with specific phenotypic changes using quantitative trait locus (QTL) analysis (Carlson et al., 2014, Protas et al., 2006). This forward genetics approach, in contrast to the reverse genetics candidate gene approaches used in zebrafish, has the ability to unbiasedly detect key factors that can distinguish between tissue regeneration and scar-based wound healing. First-generation offspring (F1) of a cross between Pachón and surface fish displayed a more surface fish-like regenerative capacity, although fewer fish had completely regenerated their myocardium at 64 dpa (Figure 1K). We further crossed two F1 siblings to obtain second-generation offspring (F2). In the F2, the parental alleles had segregated, and most chromosomes were recombinant mixtures of the parental chromosomes, resulting in offspring ranging from small, pigmented fish with no eyes to large pale fish with black eyes (Figure 5A). One hundred eighty-eight F2 fish were analyzed for their ability to regenerate fins, before being tested for heart regeneration at 90 dpa, a time point when most surface fish have regenerated. Additionally, we characterized the fish for eye size, body melanocyte number, and heart size. On the basis of wound morphology, the 90 dpa hearts were classified into seven different groups, ranging from no signs of regeneration in group 0 and completely regenerated without residual scar in group 6 (Figures 5B and 5C). These data were then linked to the other phenotypic traits. We did not find any correlation between heart regeneration and body melanocyte count (Pearson’s r = 0.085, p = 0.29, n = 155), left eye size (r = 0.089, p = 0.11, n = 171), fin regeneration (r = 0.117, p = 0.17, n = 142), or ventricle size (r = 0.113, p = 0.14, n = 171) (Figure 5D and data not shown). Even though heart regeneration is not linked to one of these traits, partial loss of heart regeneration in all tested cavefish populations suggests that this might either be beneficial to cave life or a trade-off in favor of other organ function.

DNA was isolated from the P0 surface female and Pachón male and 188 F2 fish and subsequently sequenced using a specific restriction site-associated DNA (RAD) sequencing method, creating a genome-wide genetic marker set of heritable polymorphisms (Davey et al., 2011, Hohenlohe et al., 2010). QTL analysis was performed using both the six regeneration groups (Figures 5B–5E, blue line in Figure 5E) as well as a set of continuous values, the percentage of open compact ventricular wall (Figure 5E, yellow line, open V/size V). *De novo* linkage analysis using both phenotypes identified three loci in the genome significantly linked to the degree of heart regeneration in linkage groups 1, 9, and 10 (Figure 5E). The markers within and flanking these three loci were mapped to the published cavefish genome (McGaugh et al., 2014) to identify the genes within this region (Figure 5F). Two of the three linkage groups (LGs) contained significantly differentially expressed genes between the surface fish and Pachón during heart regeneration, 7 in LG9 and 14 in LG10. None of these genes have previously been linked to heart regeneration in fish. Notably, three extracellular matrix genes, located close to the peaks of LG9 and LG10, si:dkeyp-69c1.7 (similar to Thy1), *ncam1a*, and *itgam*, are potential candidates for the difference in the scarring response between the two fish. All differentially expressed genes, as well as SNPs in either the coding sequence or enhancers, will be followed up in future functional studies to identify the most upstream causal genes regulating the decision between scarring and regeneration.

## Discussion

Although resection versus cryo-injury offers the possibility of comparing regeneration versus fibrosis in zebrafish, the latter results only in a delay of regeneration, rather than blocking it completely. Additionally, there remains the significant caveat of comparing profoundly different injuries; resection involves removal of healthy heart tissue and no scarring, whereas cryo-injury results in cellular destruction and necrosis with scar formation. Recently, the Japanese rice fish (*Oryzias latipes*), more commonly known as *Medaka*, was shown to scar after resection injury (Ito et al., 2014); however, comparison of this model with zebrafish is complicated by inter-species variations. *Astyanax mexicanus*, as a model for heart regeneration research, enables a specific focus exclusively on the mechanisms regulating heart regeneration versus scarring, from which differences in background, injury insult, and species-specific changes in response to injury have effectively been eliminated. Moreover, the ability to perform intra-species genetic analyses such as QTL comparisons between different populations of surface and cavefish is a further major advantage of the *Astyanax mexicanus* model over other model organisms.

*Medaka* lack the wound border cardiomyocyte proliferation after injury, explaining their lack of regeneration (Ito et al., 2014). In contrast, we observe similar levels of labeling between surface fish and Pachón or between wild-type and *lrrc10* knockout zebrafish, while the Pachón and zebrafish mutants do not regenerate. Pachón and *lrrc10* mutants seem to resemble the adult mammalian and human situation, whereby even though increased proliferation is observed in the myocardial cells adjacent to the injury after myocardial infarction (Senyo et al., 2013), no regeneration occurs. Interestingly, a similar process of proliferation followed by unexplained cell loss was observed in the adult Pachón retina (Strickler et al., 2007). Although it is possible that the cardiomyocytes either die via a caspase-independent pathway or re-differentiate into a non-myocardial cell type, the fact that similar numbers of BrdU-positive cells are present at 14 dpa compared with 7 dpa points to defective nuclear division. If proliferating Pachón cardiomyocytes bordering the injury indeed fail cytokinesis and as a result do not regenerate, this would make the cavefish a model to understand the molecular mechanisms regulating polyploidization versus proliferation after injury. The non-regenerative response in mammals has been attributed to the high level of cardiomyocyte polyploidy, whereas fish cardiomyocytes are normally diploid and are thus more capable of proliferation. Genetically induced polyploidy in the zebrafish heart has been shown to block regeneration (González-Rosa et al., 2018, Patterson et al., 2017).

In both Pachón and *lrrc10*-mutant zebrafish, high global myocardial proliferation is observed, albeit at 30 dpa in Pachón compared with 7dpi in *lrrc10* mutants, suggesting that also the global proliferation normally observed in zebrafish after injury is not directly linked to heart regeneration. The late increase in endocardial proliferation on the basal side of the Pachón hearts at 14 dpa might be linked to the increase observed in myocardial proliferation at 30 dpa. Signals from the endocardium after injury in zebrafish are required for cardiomyocyte proliferation and regeneration (Kikuchi et al., 2011). However, this increase in late global proliferation does not rescue regeneration and could even be detrimental to the process.

Combined with our transcriptome analysis, our data therefore affirms that also in fish, repair and regeneration is a multifactorial process, with scar formation and regression acting as a critical determinant on a par with cardiomyocyte proliferation. Further detailed analysis of the interplay between myocardial proliferation, fibrosis, and the immune response as well as the three loci linked to the ability for heart regeneration, discovered herein, should enable the identification of factors that underpin tissue regeneration versus fibrosis, scar formation, and scar regression. Extrapolating from *Astyanax mexicanus* might provide clues as to why adult mammals lost the ability to regenerate their hearts during evolution and may ultimately lead to strategies to promote optimal human heart repair.

## STAR★Methods

### Key Resources Table

**Table:** 

REAGENT or RESOURCE	SOURCE	IDENTIFIER
**Antibodies**		

Goat polyclonal against Cardiac Troponin I	Hytest	Cat# 4T21/2; RRID:AB_154084
Rabbit polyclonal against Myocyte Enhancer Factor 2	Santa Cruz Biotechnology	Cat# sc-313; RRID:AB_631920
Anti-Cleaved Caspase-3	Cell Signaling Technology	Cat# 9661; RRID:AB_2341188
Monoclonal mouse against 5-bromo-2′-deoxyuridine (BrdU) (BU5.1)	Progen	Cat# 03-61015; RRID:AB_1540350
Monoclonal mouse against Proliferating Cell Nuclear Antigen antibody (PCNA clone PC10)	Dako	Cat# M0879; RRID:AB_2160651
Monoclonal mouse against Myosin Heavy Chain (MF20)	Developmental Studies Hybridoma Bank	Cat# MF 20; RRID:AB_2147781

**Chemicals, Peptides, and Recombinant Proteins**		

Acid Fuchsin Orange-G (AFOG)	Poss et al., 2002	N/A
MS222	Sigma	Cat# A5040; CAS: 886-86-2
4% paraformaldehyde (PFA) in phosphate-buffered saline (PBS)	Santa Cruz	Cat# sc-281692 CAS: 30525-89-4
TNB block	Perkin Elmer	Cat# NEL702001KT
ERCC RNA Spike-In Control Mixes	Ambion	Cat# 4456740
Fast SYBR Green Master Mix	Life technologies	Cat# 4385612
Cas9 nuclease 3NLS protein	IDT	Cat# 1081058
SbfI restriction enzyme	New England Biolabs	Cat# R0642S

**Critical Commercial Assays**		

TSA fluorescein kit	Perkin Elmer	Cat# NEL756001KT
TSA Plus Cyanine 3 system	Perkin Elmer	Cat# NEL744001KT
TSA Plus TMR System	Perkin Elmer	Cat# NEL742001KT
RNAscope Multiplex Fluorescent Reagent Kit v2	Advanced Cell Diagnostics	Cat# 323100
RNeasy Mini Plus kit	QIAGEN	Cat# 74134
ScriptSeq v2 RNA-Seq Library Preparation Kit	Epicenter	Cat# SSV21106
QuantiTect Whole Transcriptome kit	QIAGEN	Cat# 207043
QIAquick purification	QIAGEN	Cat# 28104
MEGAshortscript T7 Transcription Kit	Ambion	Cat# AM1354
DNeasy Blood & Tissue Kit	QIAGEN	Cat# 69504

**Deposited Data**		

RNA sequencing - Raw sequence files deposited to European Nucleotide Archive	This paper	ENA: PRJEB26684
Astyanax mexicanus genome - AstMex102 & transcript annotations v84(GCA_000372685.1)	Ensembl	www.ensembl.org/Astyanax_mexicanus/Info/Index
Gene Ontology (GO) annotations (including zebrafish homology)	Ensembl Biomart	https://www.ensembl.org/biomart/martview
Zebrafish symbol mappings of GO gene signatures for Gene Set Enrichment Analysis (GSEA)	Subramanian et al., 2005	http://www.bioinformatics.org/go2msig/April 2015 version
QTL - Raw sequence files deposited to the European Nucleotide Archive	This paper	ENA: PRJEB26692

**Experimental Models: Organisms/Strains**		

Zebrafish Wild type (AB)	ZIRC	ZFIN ID: ZDB-GENO-960809-7
Zebrafish Mut: *lrrc10 −/−*	This paper	N/A
Astyanax Mexicanus: Surface fish	This paper	N/A
Astyanax Mexicanus: Pachón	This paper	N/A
Astyanax Mexicanus: Tinaja	This paper	N/A
Astyanax Mexicanus: Chica	This paper	N/A
Astyanax Mexicanus: F1/F2 surface fish x Pachón hybrids	This paper	N/A

**Oligonucleotides**		

SgRNA: GGTTTAGGTATCCGAAAGCAGG	This paper	N/A
SgRNA: TTCCAGTCGCCCGAGCTCGG	This paper	N/A
Lrrc10 genotyping FW primer: 5′-GTAACGTGTTTCCTG ATGCCA-3′	This paper	N/A
Lrrc10 genotyping RV primer: 5′-CTGACAAATGCGAT TGCGGT-3′	This paper	N/A
lrrc10 riboprobe FW 5′-GCCAAGAAGATGGGAAAT GTTG-3′	This paper	N/A
lrrc10 riboprobe RV 5′-AGTGTTCACCGCAGCTTT-3′	This paper	N/A
See Table S1 for qPCR primers	This paper	N/A

**Software and Algorithms**		

ImageJ	Schneider et al., 2012	https://imagej.net/RRID:SCR_003070
Trimmomatic v.0.36	Bolger, A. M., Lohse, M., & Usadel, B., 2014	http://www.usadellab.org/cms/?page=trimmomatic RRID:SCR_011848
FastQC v0.11.3	Babraham Institute	http://www.bioinformatics.babraham.ac.uk/projects/fastqc/RRID:SCR_014583
STAR v.2.5.2a (single pass mode)	Dobin et al., 2013	https://github.com/alexdobin/STAR
Burrows-Wheeler Aligner - BWA (v.0.7.12-r1039)	Li and Durbin., 2009	http://bio-bwa.sourceforge.net/RRID:SCR_010910
R Project	R Core Development Team, 2015	http://www.R-project.org/RRID:SCR_001905
edgeR	Robinson et al., 2010	https://bioconductor.org/packages/release/bioc/html/edgeR.html RRID:SCR_012802
RUVg	Risso et al., 2014	https://bioconductor.org/packages/release/bioc/html/RUVSeq.html
DESeq2	Love et al., 2014	https://bioconductor.org/packages/release/bioc/html/DESeq2.html RRID:SCR_015687
Short Time-series Expression Miner (STEM)	Ernst and Bar-Joseph., 2006	http://www.cs.cmu.edu/∼jernst/stem/RRID:SCR_005016
Pretty Heatmaps. R package version 1.0.8.	Kolde, 2015	http://CRAN.R-project.org/package=pheatmap
CCTop	Haeussler et al., 2016	https://crispr.cos.uni-heidelberg.de/
CRISPOR	Stemmer et al., 2015	http://crispor.tefor.net/RRID:SCR_015935
Stacks	Catchen et al., 2011	http://catchenlab.life.illinois.edu/stacks/
R/qtl	Broman et al., 2003	http://www.rqtl.org/RRID:SCR_009085
BLASTN	Altschul et al., 1990	https://blast.ncbi.nlm.nih.gov/Blast.cgi RRID:SCR_001598

**Other**		

Illumina HiSeq 2000 (2x100bp paired-end protocol) – RNA sequencing.	Exeter Sequencing Service	N/A
Illumina HiSeq 2000 (single-end 100 bp chemistry) – RAD fragment library sequencing.	Exeter Sequencing Service	N/A
RNAscope *lrrc10* probe (Zebrafish)	Advanced Cell Diagnostics	Cat# 548361-C3
RNAscope *lrrc10* probe (Astyanax Mexicanus)	Advanced Cell Diagnostics	Cat# 532011-C2

### Contact for Reagent and Resource Sharing

Further information and requests for resources and reagents should be directed to and will be fulfilled by the Lead Contact, Mathilda Mommersteeg (mathilda.mommersteeg@dpag.ox.ac.uk).

### Experimental Model and Subject Details

#### Animals

All experimental procedures were performed in accordance with the UK Animals (Scientific Procedures) Act 1986 and institutional guidelines and conform to the guidelines from Directive 2010/63/EU of the European Parliament on the protection of animals used for scientific purposes. Adult *Astyanax mexicanus* surface fish, Pachón, Tinaja, Chica, and F1/F2 surface fish x Pachón hybrids were bred and maintained in the laboratory at 22–25°C on a 14/10-hour photo-period. All *Danio rerio* zebrafish and embryos were bred and maintained at 28°C on a 12/12-hour photo-period. Both male and female fish were used for experiments.

### Method Details

#### Cardiac injury

Prior to all surgical operations, fish were anaesthetized in MS222 (Sigma). Apical resections were performed as previously described (Poss et al., 2002). Briefly, spring scissors were used to make a small incision to penetrate the thorax and open the pericardial sac. The ventricle was exposed and the most apical tip of the ventricle was removed by scissors. Bleeding was stopped by applying pressure to the wound using tissue paper. Cryoinjury in zebrafish was performed as previously described (González-Rosa et al., 2011). A small incision was made through the thorax and the pericardium using forceps and spring scissors. The abdomen was gently squeezed to expose the ventricle and tissue paper was used to dry the heart. A cryo-probe with a copper filament was cooled in liquid nitrogen and placed on the ventricle surface until thawing was observed. Body wall incisions were not sutured, and after surgery, fish were returned to water and stimulated to breathe by pipetting water over the gills until the fish started swimming again. For sham surgery, the thorax and pericardial sac were opened, but the heart was not injured. Hearts were isolated at the indicated different time points, processed for RNA isolation or fixed overnight at 4°C in 4% paraformaldehyde (PFA) in phosphate-buffered saline (PBS), before dehydration and paraffin embedding.

#### Fin resection and analysis

The fish were anaesthetised in MS222. The tail fin was imaged before removing the dorsal lobe using a sharp scissor. After resection, the fin was imaged again before the fish was returned to the tank water. After 14 days, the fish were anaesthetised and the regrowth of the fin was imaged before returning the fish to the water. Fin regeneration was quantified by measuring the length of the dorsal fin lobe from the body to the resection line, which was subtracted from the length of the ventral uncut fin lobe to obtain the length of the removed fin. The length of the regenerated dorsal fin lobe was measured from the resection line and expressed as percentage of the removed fin. All measurements were performed in ImageJ (Schneider et al., 2012) and lengths were measured following 3 different fin rays to determine the longest ray, which was used for analysis. The blood vessel at the base of the fin was used as reference point for measurements starting from the body.

#### BrdU labeling

For the BrdU labeling experiments, animals were exposed to 10mM 5-bromo-2′-deoxyuridine (BrdU, Sigma) in tank water for 24 hours at 6 dpa or 13 dpa, returned to their tanks and the hearts isolated after 24 hours.

#### Histology

7-12μm paraffin sections were mounted on Superfrost slides and dried overnight at 37°C. Sections were deparaffinised in Histoclear (National Diagnostics), rehydrated and washed in distilled water. Acid Fuchsin Orange-G (AFOG) staining was performed as described previously (Poss et al., 2002), staining myocardium orange, fibrin red and collagen bright blue.

#### Immunohistochemistry

Fluorescent immunohistochemistry was performed as previously described (Mommersteeg et al., 2010). Paraffin sections of 7 μm thick were pressure cooked for 4 minutes in Antigen unmasking solution (H-3300, Vector Laboratories Inc) after deparaffinisation and rehydration. After cooling down, the sections were blocked using TNB (NEL702001KT, Perkin Elmer) followed by primary antibody overnight in TNB. The following primary antibodies were used: goat polyclonal against Cardiac Troponin I (cTnI, 1:200, Hytest, 4T21/2), rabbit polyclonal against Myocyte Enhancer Factor 2 (Mef2 C-21, 1:200, Santa Cruz, sc-313), Cleaved Caspase-3 (CC3 Asp175, 1:200; Cell Signaling Technology, 9661S), and mouse monoclonal antibodies against 5-bromo-2′-deoxyuridine (BrdU BU5.1, 1:200. Progen, 61015), Proliferating Cell Nuclear Antigen (PCNA clone PC10, 1:200, Dako Cytomation, M0879) and Myosin Heavy Chain (MF20, 1:50, Developmental Studies Hybridoma Bank). All antibodies are commonly used in zebrafish. For PCNA/BrdU and Mef2 double labeling, after washing in PBS-T, sections were incubated with Alexa 488 goat anti-mouse and 546 goat anti-rabbit antibodies (1:200, Molecular Probes) for 2 hours. For double labeling of cTnI and CC3, sections were processed with the TSA fluorescein kit (NEL756001KT, Perkin Elmer). Alexa 546 donkey anti-goat (1:200, Molecular Probes) was combined with biotinylated horse anti-rabbit (1:200, Vector Laboratories Inc, BA-1100) for 2 hours. After washing in PBS-T, sections were incubated with streptavidin-HRP antibody for 30 minutes (1:200), washed again and stained with Fluorescein (NEL756001KT, Perkin Elmer LAS). For the counter stain with MF20 following RNAscope *in situ* hybridization, sections were incubated with Alexa 488 goat anti-mouse (1:200, Invitrogen) for 2 hours. After washing, the sections were mounted in Mowiol 4-88 (Applichem). Nuclei were counterstained with DAPI (2.5 μg/ml;Sigma). Images were processed in ImageJ to generate magenta and green color combinations.

#### RNAscope *in situ* hybridization

RNAscope (Advanced Cell Diagnostics, Hayward, CA) was performed on 7μm thick paraffin sections, previously fixed in 4% PFA in PBS. Sections were deparaffinised and were boiled (98-102°C) with RNAscope target retrieval for 15 min followed by incubation with RNAscope protease III at 40°C for 15 min. Following protease treatment, sections were incubated with the *lrrc10* probe for 2 hours at 40°C. To detect the hybridization signal, RNAscope Multiplex Fluorescent Detection Reagents v2 utilizing the TSA Plus Cyanine 3 fluorophore (Perkin Elmer, NEL744001KT) were applied according to the manufactures instructions. Advanced Cell Diagnostics designed the *lrrc10* probe. Sections were counterstained with anti-MF20 (see immunohistochemistry).

#### Wound length measurements

For assessment of regenerative capacity, paraffin sections were prepared through the entire ventricle and stained using AFOG staining. Using ImageJ, the width of the wound was measured from compact myocardial wall to compact myocardial wall on the section with the largest opening. Additionally, the ventricular perimeter was measured at the largest part of the ventricle. Percentage of regeneration was expressed as wound width divided by ventricle perimeter x100.

#### Proliferation counts

Proliferation counts near the injury site were performed using both PCNA and BrdU. To correct for large differences in wound size, especially comparing surface fish and Pachón at 30 dpa, we counted the number of PCNA or BrdU and Mef2 positive nuclei surrounding the injury site and divided this number by the length of the wound border in μm. The 3 sections with the largest wound border length per heart were counted. For proliferation counts on the basal side of the ventricle, a square area of 0.103mm^2^ was measured, which included both compact wall and trabecular areas. Per section, one square was counted with 5 sections counted per heart.

#### RNA sequencing and analysis

For RNA sequencing, one year old surface fish and Pachón hearts were isolated at 3, 7 and 14 dpa. For sham surgery, the thorax and pericardial sac were opened, but the heart was not injured and the heart was isolated 3 days later. Directly after isolation, all ventricles were stored separately in 500μl RNAlater (QIAGEN) until further processing. Three ventricles were combined per sample, with three different samples per time point for both surface fish and Pachón. The ventricles were transferred to a new tube containing RLT buffer and 10 matrix D beads (MP Biomedicals) and lysed using the FastPrep FP120 tissue lyser. RNA was extracted using the RNeasy Mini Plus kit (QIAGEN) and analyzed using the Experion Automated Electrophoresis System (Biorad). The samples were spiked-in with ERCC RNA Spike-In Control Mixes (2ul of 1:100, Ambion), prepared for RNA sequencing using the ScriptSeq v2 RNA-Seq Library Preparation Kit (Epicenter) and sequenced using the Illumina HiSeq 2000 (2x100bp paired-end protocol; Exeter Sequencing Service). Raw sequence files have been deposited in the European Nucleotide Archive (ENA: PRJEB26684). Reads were trimmed with Trimmomatic (v.0.36, http://www.usadellab.org/cms/?page=trimmomatic) (Bolger et al., 2014) and controlled for quality with FastQC (v0.11.3, http://www.bioinformatics.bbsrc.ac.uk/projects/fastqc) before alignment to the *Astyanax mexicanus* genome (AstMex102 downloaded from Ensembl along with transcript annotations v84). Reads were aligned using STAR in single-pass mode (v.2.5.2a, https://github.com/alexdobin/STAR) (Dobin et al., 2013) with standard parameters but specifying “alignIntronMax 100000.” Unmapped reads were aligned to ERCC spike-in sequences with BWA (v.0.7.12-r1039) (Li and Durbin, 2009). The ERCC counts were added to the transcript counts for subsequent normalization. Only transcripts (including spike-ins) with > 5 reads in > 2 samples were retained for further processing. The filtering step removed 13/92 spike-ins (14%) and 7,062/23,772 transcripts (30%). Raw counts were loaded into R (http://www.R-project.org/) (R Core Development Team, 2015) and edgeR (Robinson et al., 2010) was used to perform upper quantile, between-lane normalization before RUVg (Risso et al., 2014) was run to model unwanted variation based on the spike-ins. Subsequent differential expression analyses included the “W_1” RUVg matrix in the model. Between-lane normalization, upper quartile normalization and dispersion estimates were carried out as recommended in the edgeR documentation. Values generated with the rpkm function of edgeR, including library size normalization and log2 conversion, were used in figures. edgeR was used for pairwise comparisons and DESeq2 (Love et al., 2014) was used for time series. Gene Ontology (GO) annotations (including zebrafish homology) were retrieved from the Ensembl Biomart. Zebrafish symbol mappings of GO gene signatures for Gene Set Enrichment Analysis (GSEA) (Subramanian et al., 2005) were retrieved from http://www.bioinformatics.org/go2msig/ (April 2015 version). Pre-ranked GSEA was run using log2 fold change as the ranking metric. Short Time-series Expression Miner (STEM) (Ernst and Bar-Joseph, 2006) was used to cluster time series profiles (default parameters except for using FDR and excluding GO evidence codes NAS, NR and ND). Heatmaps were generated using pheatmap (Pretty Heatmaps. R package version 1.0.8. http://CRAN.R-project.org/package=pheatmap) (Kolde, 2015). Other plots were made using in-house R scripts (available upon request).

#### Quantitative PCR

For quantitative PCR, a new set of one year old surface fish and Pachón hearts were isolated at 7 dpa. Directly after isolation, all ventricles were stored separately in 500μl RNAlater (QIAGEN) until further processing. RNA was isolated from 3 different samples with one ventricle per sample for both surface fish and Pachón using the RNeasy Mini Plus kit (QIAGEN). cDNA was produced and amplified using the QuantiTect Whole Transcriptome kit (QIAGEN). Quantitative PCR (qPCR) was performed using the ViiA7 (Applied Biosystems, Life Technologies) and Fast SYBR Green Master Mix (Life technologies). Samples contained 1x Fast SYBR Green Master Mix, 150 – 400nM of each primer set and 5 μL cDNA (1:1000 dilution of RT) for a final reaction volume of 25 μl. Primer sets for the genes of interest were used at a 200nM concentration, and the primer sets for the housekeeping genes, *rpl13a* and *18 s*, were used at 400nM and 150nM respectively. Samples were run in triplicate in optically clear fast 96-well plates (Applied Biosystems, Life Technologies) and each qPCR run was repeated a second time. Thermocycling parameters consisted of a holding step for 20 s at 95°C, followed by 40 cycles of 95°C for 1 s and 60°C for 20 s. For each sample a melt curve step was performed at 95°C for 15 s, 60°C for 1 minute, followed by 95°C for 15 s at the end of the amplification stage, in order to identify a specific melting peak for each primer set. The relative expression of each gene was determined after normalization to the mean of the housekeeping genes, *rpl13a* and *18 s*, using the −ΔΔCT method. The relative expression of each gene was calculated relative to the surface fish expression.

#### CRISPR mediated generation of zebrafish lrrc10 mutants

*Lrrc10* ^*−/−*^ mutants were generated using commercially available Cas9 nuclease 3NLS protein (IDT) and two sgRNAs. The two sgRNA target sites were selected using CCTop and CRISPOR (Haeussler et al., 2016, Stemmer et al., 2015) and were designed to target the first intron and the second exon, excising 601bp from the gene (sgRNA target site intron: GGTTTAGGTATCCGAAAGCAGG and sgRNA target site exon 2: TTCCAGTCGCCCGAGCTCGGCGG (PAMs underlined)). The sgRNAs were generated as previously described using *in vitro* transcription of an oligo-based method (Gagnon et al., 2014). In brief, sgRNAs were made using oligonucleotides containing the T7 promoter sequence, the sgRNA target site and a complementary region, which were annealed to a constant oligonucleotide encoding the tracrRNA. Oligo overhangs were extended using T4 DNA polymerase (NEB) followed by QIAquick purification (QIAGEN). The resulting dsDNA product was subsequently used as the template for sgRNA *in vitro* transcription using the MEGAshortscript T7 Transcription Kit (Ambion), followed by DNase treatment and precipitation with ammonium actetate/ethanol. Gene specific oligo: 5′-TAATACGACTCACTATAGG -N20-GTTTTAGAGCTAGAAATAGCAAG-3′ (-N20- is the sgRNA target sequence) Constant oligo: 5′-AAAAGCACCGACTCGGTGCCACTTTTTCAAGTTGATAACGGACTAGCCTTATTTTAACTTGCTATTTCTAGCTCTAAAAC-3′. The injection mixture contained 800ng/μl cas9 protein, 80ng/μl each sgRNA and 300mM KCl. The injection mixture was incubated at 37°C for 5min and 1nl of the mixture was microinjected into the one cell stage of the zebrafish embryo. Mutant alleles were identified by PCR amplification and sequencing using the forward primer: 5′-GTAACGTGTTTCCTGATGCCA-3′ and the reverse primer: 5′-CTGACAAATGCGATTGCGGT-3′. The wild-type product is 833bp, whereas the mutant product is 232bp. Fish were bred to F2 generation to generate homozygous *lrrc10* ^*−/−*^. Whole mount *in situ* hybridization using an *lrrc10* antisense probe in *lrrc10* ^*−/−*^ and wild-type embryos showed no signal in the mutant compared to the wild-type, confirming knockout of *lrrc10.* The primer pairs used to generate DIG-labeled both sense and antisense lrrc10 riboprobes were as the followings: lrrc10 FW 5′-GCCAAGAAGATGGGAAATGTTG-3′ and lrrc10 RV 5′-AGTGTTCACCGCAGCTTT-3′.

#### F2 heart regeneration and correlation analysis

The extent of heart regeneration was determined using AFOG staining on 12μm sections in 188 one year old F2 fish at 90 dpa using 2 different measurements. For the first measurement, fish were divided into 7 categories based on morphology of the wound. Group 0, clear scar, neither compact wall myocardial thickening nor overgrowth (arrowhead). Group 1, clear scar, no compact wall overgrowth, but thickening of the compact wall directly adjacent to the scar. Group 2, clear scar, compact myocardium has started to grow over the scar (arrowhead). Group 3, clear scar, compact wall has grown over a large part of the scar, but the edges have not yet met. Group 4, clear scar, compact myocardium closed. Group 5, very small scar left, compact myocardium closed. Group 6, no sign of injury left. As an additional method the number of sections was determined in which the compact wall of the ventricle had not closed, expressed as percentage of the total amount of sections per ventricle. Fin regeneration was measured as described above. Ventricle size was determined by the number of 12μm sections per ventricle. To assess body pigmentation, a box of the same size was placed on equivalent dorsal regions of images of all F2 fish. The number of pigment cells within the box was then counted. Correlation was determined using Pearson’s correlation.

#### Quantitative Trait Locus (QTL) analysis

For RAD-sequencing, DNA was isolated from fins of the 188 F2 fish as well as from the P0 surface female and Pachón male using the DNeasy Blood & Tissue Kit (QIAGEN). Library preparation was carried out by Floragenex (Eugene, Oregon, USA) following the protocol of Etter et al. (Etter et al., 2011). Briefly, genomic DNA was digested with SbfI (New England Biolabs) and libraries from individual F2 fish were barcoded. After random shearing with a Bioruptor (Diagenode), DNA 250 bp to 500 bp in size was isolated and RAD fragment libraries were sequenced on an Illumina HiSeq 2000 using single-end 100 bp chemistry. Raw sequence files have been deposited in the European Nucleotide Archive (ENA: PRJEB26692). FASTQ sequence data were demultiplexed and trimmed to 91 bp. The Stacks (v.1.44) (Catchen et al., 2011) function process_radtags was used to remove poor quality reads. The remaining reads (2.6 million/sample on average) were processed with Stacks to identify single nucleotide polymorphisms (SNPs) and genotype F2 fish at these SNPs, essentially as described in the Stacks *de novo* pipeline documentation. Stack formation used default parameters except for requiring a minimum depth of 3 and enabling repetitive stack removal (ustacks -r -m 3). The stacks catalog was built using the P0 fish, allowing 2 mismatches (cstacks -n 2). Each fish was then matched to this catalog with sstacks. For QTL analysis, 176 F2 fish with phenotype data were used. The Stacks MySQL database interface was used to filter tags to retain only those where P0 fish had different, homozygous alleles and at least 150/176 F2 fish were genotyped. Tags were also filtered on log likelihood (lnl > −10). The genotypes for the 6,845 resulting markers were formatted along with phenotype values for import into R/qtl (Broman et al., 2003) for QTL analysis. We next excluded markers with distorted segregation patterns (p value < 0.05/6,845). Genotypes for the remaining 5,634 markers were found in the expected 1:2:1 ratio (AA:26.3%, AB:49.2%, BB:24.4%). Linkage groups (LG) were formed with maximum recombination fraction (RF) = 25% and minimum LOD = 6.9. Three LG with only 2 markers each were removed. After rippling and manual rearrangement to maximize order LOD score and minimize length, one LG was split into two as it consisted of two distinct blocks with high RF and low LOD between the blocks. The final set of 25 LG was scanned for markers linked with regeneration category or with percent open compact ventricular wall (open V / size V). The genome-wide LOD significance threshold was set at the 95th percentile of 1,000 permutations. Markers on LG with LOD peaks (LG 1, 9, 10) were aligned to the cavefish genome using BLASTN (Altschul et al., 1990). Single best hits were retained if they mapped the full length of the read with > 95% perfect nucleotide matches. Unaligned markers (∼10%) were dropped from these 3 LG. Remaining markers were then rearranged where necessary to keep the mapped contig order, even at the cost of reducing overall LOD and increasing length. Five markers from LG1 mapped to the same contig as 11 LG9 markers so the 5 were moved to LG9. QTL scans were repeated with this post-BLAST arrangement and identified the same high LOD regions. For the Circos (Krzywinski, 2009) plots, LG were scaled to make LG1 roughly the same size as the largest contig shown. LOD score tracks show values from the QTL scans using the post-BLAST genetic map. Marker positions within and flanking the high LOD regions were linked to the midpoint of their aligned position on the cavefish contigs. For expression log2 fold change (logFC) tracks, transcripts overlapping the linked contig regions were identified in our RNA-seq data. LogFC values are shown at the midpoint of the corresponding gene.

### Quantification and Statistical Analysis

The number of samples (n) used in each experiment is shown in the legends and recorded in detail below. Gene expression experiments have been done 3 times independently. ANOVA tests were applied when normality and equal variance tests were passed. Results are expressed as mean ± SEM (^∗^ for p < 0.05, ^∗∗^ for p < 0.01, ^∗∗∗^ for p < 0.001 and ^∗∗∗∗^ for p < 0.0001). Statistical analysis was performed in GraphPad Prism 6 for Windows, GraphPad Software, La Jolla California USA, www.graphpad.com.

Figure 1G. 0 dpa: surface n = 6, Pachón n = 5, p = 0.9996. 7 dpa: surface n = 5, Pachón n = 4, p > 0.999. 14 dpa: surface n = 4, Pachón n = 4, p = 0.0124. 21 dpa surface n = 5, Pachón n = 3, p = 0.1038. 30 dpa: surface n = 7, Pachón n = 5, p < 0.0001. 50-53 dpa: surface n = 3, Pachón n = 5, p = 0.0004. 64 dpa: surface n = 10, Pachón n = 7, p < 0.0001. Pachón 100 dpa n = 5, 365 dpa n = 3. Two-way ANOVA with Sidak’s multiple comparisons test.

Figure 1H. Pachón (n = 18), surface fish (n = 16). Unpaired, two-tailed, unequal variance t test, p = 0.1674.

Figure 1K. surface fish (n = 10, versus Pachón p = 0.0002, versus Tinaja p = 0.1115, versus Chica p = 0.2372, versus F1 p = 0.9828), Pachón (n = 7, versus Tinaja p = 0.2221, versus Chica p = 0.0546, versus F1 p = 0.0066), Tinaja (n = 6, versus Chica p = 0.9809, versus F1 p = 0.4670), Chica (n = 8, versus F1 p = 0.7220), surface fish x Pachón F1 hybrids (n = 5). One-way ANOVA with Tukey’s multiple comparisons test.

Figure 2A. 3 dpa: surface fish, n = 4, Pachón n = 4, p = 0.9598. 7 dpa: surface n = 5, Pachón n = 4, p = 0.3632. 14 dpa: surface n = 4, Pachón n = 4, p = 0.4528. 30 dpa: surface n = 4, Pachón n = 4, p > 0.9999. Two-way ANOVA with Sidak’s multiple comparisons test. 3 dpa versus 7 dpa: surface and Pachón p < 0.0001. 3 dpa versus 14 dpa: surface p = 0.0140, Pachón p = 0.1191. 3 dpa versus 30 dpa: surface p = 0.8794, Pachón p = 0.4248. 7 dpa versus 14 dpa: surface, p = 0.0002, Pachón p = 0.0004. 7 dpa versus 30 dpa: surface and Pachón p < 0.0001. 14 dpa versus 30 dpa: surface p = 0.1601, Pachón p = 0.9845. Two-way ANOVA with Tukey’s multiple comparisons test.

Figure 2C. 7 dpa, surface fish (n = 4), Pachón (n = 5). 14 dpa, surface fish (n = 6), Pachón (n = 6). One-way ANOVA with Tukey’s multiple comparisons test: surface 7 dpa versus Pachón 7 dpa p = 0.9987, surface 7 dpa versus surface 14 dpa p = 0.0004, surface 7 dpa versus Pachón 14 dpa p = 0.9966, Pachón 7 dpa versus surface 14 dpa p = 0.0001, Pachón 7 dpa versus Pachón 14 dpa p = 0.9792, Surface 14 dpa versus Pachón 14 dpa p = 0.0002.

Figure 2F. surface fish, 3 dpa n = 5, 7 dpa n = 5, 14 dpa n = 4, 30 dpa n = 4. Pachón, 3 dpa n = 5, 7 dpa n = 4, 14 dpa n = 4, 30 dpa n = 4. surface versus Pachón: 3 dpa p = 0.0033, 7 dpa p = 0.0194, 14 dpa p = 0.0009, 30 dpa p = 0.1257. Two-way ANOVA with Sidak’s multiple comparisons test. 3 dpa versus 7 dpa: surface p = 0.8685, Pachón p = 0.9243. 3 dpa versus 14 dpa: surface p = 0.0066, Pachón p = 0.1754. 3 dpa versus 30 dpa: surface p = 0.0012, Pachón p < 0.0001. 7 dpa versus 14 dpa: surface, p = 0.0705, Pachón p = 0.5434. 7 dpa versus 30 dpa: surface p = 0.0226, Pachón p = 0.0013. 14 dpa versus 30 dpa: surface p = 0.9843, Pachón p = 0.0353. Two-way ANOVA with Tukey’s multiple comparisons test.

Figure 2H. surface fish, 3 dpa n = 5, 7 dpa n = 5, 14 dpa n = 4, 30 dpa n = 4. Pachón, 3 dpa n = 5, 7 dpa n = 4, 14 dpa n = 4, 30 dpa n = 4. surface versus Pachón: 3 dpa p = 0.9983, 7 dpa p > 0.9999, 14 dpa p < 0.0001, 30 dpa p = 0.9229. Two-way ANOVA with Sidak’s multiple comparisons test. 3 dpa versus 7 dpa: surface p = 0.9786, Pachón p = 0.9166. 3 dpa versus 14 dpa: surface p = 0.8227, Pachón p < 0.0001. 3 dpa versus 30 dpa: surface p = 0.9955, Pachón p = 0.6040. 7 dpa versus 14 dpa: surface, p = 0.9601, Pachón p < 0.0001. 7 dpa versus 30 dpa: surface p = 0.9990, Pachón p = 0.9631. 14 dpa versus 30 dpa: surface p = 0.9312, Pachón p < 0.0001. Two-way ANOVA with Tukey’s multiple comparisons test.

Figure 2J. surface fish, 3 dpa n = 5, 7 dpa n = 5, 14 dpa n = 4, 30 dpa n = 4. Pachón, 3 dpa n = 5, 7 dpa n = 4, 14 dpa n = 4, 30 dpa n = 4. surface versus Pachón: 3 dpa p = 0.6125, 7 dpa p > 0.9999, 14 dpa p = 0.9785, 30 dpa p = 0.0003. Two-way ANOVA with Sidak’s multiple comparisons test. 3 dpa versus 7 dpa: surface p = 0.9329, Pachón p = 0.9470. 3 dpa versus 14 dpa: surface p = 0.5507, Pachón p = 0.9764. 3 dpa versus 30 dpa: surface p = 0.3629, Pachón p < 0.0001. 7 dpa versus 14 dpa: surface, p = 0.8658, Pachón p = 0.9985. 7 dpa versus 30 dpa: surface p = 0.1435, Pachón p < 0.0001. 14 dpa versus 30 dpa: surface p = 0.0402, Pachón p < 0.0001. Two-way ANOVA with Tukey’s multiple comparisons test.

Figure 2L. surface fish, 3 dpa n = 5, 7 dpa n = 5, 14 dpa n = 4, 30 dpa n = 4. Pachón, 3 dpa n = 5, 7 dpa n = 4, 14 dpa n = 4, 30 dpa n = 4. surface versus Pachón: 3 dpa p = 0.9577, 7 dpa p = 0.9705, 14 dpa p = 0.5651, 30 dpa p = 0.0692. Two-way ANOVA with Sidak’s multiple comparisons test. 3 dpa versus 7 dpa: surface p = 0.7846, Pachón p = 0.8585. 3 dpa versus 14 dpa: surface p = 0.5278, Pachón p = 0.9530. 3 dpa versus 30 dpa: surface p = 0.3324, Pachón p = 0.0002. 7 dpa versus 14 dpa: surface, p = 0.9614, Pachón p = 0.9907. 7 dpa versus 30 dpa: surface p = 0.8357, Pachón p = 0.0066. 14 dpa versus 30 dpa: surface p = 0.9869, Pachón p = 0.0014. Two-way ANOVA with Tukey’s multiple comparisons test.

Figure 2O. both surface fish and Pachón n = 4. Unpaired, two-tailed, equal variance t test, p = 0,0274.

Figure 3E. qPCR, surface fish n = 3, Pachón n = 3. Two-tailed unpaired t test. zgc:172270/Caveolin p = 0.0011. lrrc10 p = 0.0219. sfrp2 p = 0.0116. snai1b p = 0.0111. ckba p = 0.0095. xpo1a p = 0.0198.

Figure 4G. wild-type fish n = 7, *lrrc10*^*−/−*^ n = 7. Unpaired, two-tailed, unequal variance t test, p = 0.0140.

Figure 4I. wild-type fish n = 6, *lrrc10*^*−/−*^ n = 5. Unpaired, two-tailed, equal variance t test, p = 0.1552.

Figure 4K. wild-type fish n = 6, *lrrc10*^*−/−*^ n = 5. Unpaired, two-tailed, equal variance t test, p = 0.0008.

### Data and Software Availability

The accession number for the RNA-seq data files reported in this paper is ENA: PRJEB26684. The accession number for the RAD-seq data files reported in this paper is ENA: PRJEB26692.

## Supplemental Information

Document S1. Figures S1 and S2 and Table S1

Document S2. Article plus Supplemental Information
