# Supplementary material for: Heart Regeneration in the Mexican Cavefish
Source: Cell Rep. 2018 Nov 20;25(8):1997–2007.e7. doi: 10.1016/j.celrep.2018.10.072 (PMC6280125; doi:10.1016/j.celrep.2018.10.072)
Supplement: Document S1. Figures S1 and S2 and Table S1 [file mmc1.pdf]

**Cell Reports, Volume *25***

## Supplemental Information

# Heart Regeneration in the Mexican Cavefish

**William T. Stockdale, Madeleine E. Lemieux, Abigail C. Killen, Juanjuan Zhao, Zhilian Hu, Joey Riepsaame, Noémie Hamilton, Tetsuhiro Kudoh, Paul R. Riley, Ronny van Aerle, Yoshiyuki Yamamoto, and Mathilda T.M. Mommersteeg**

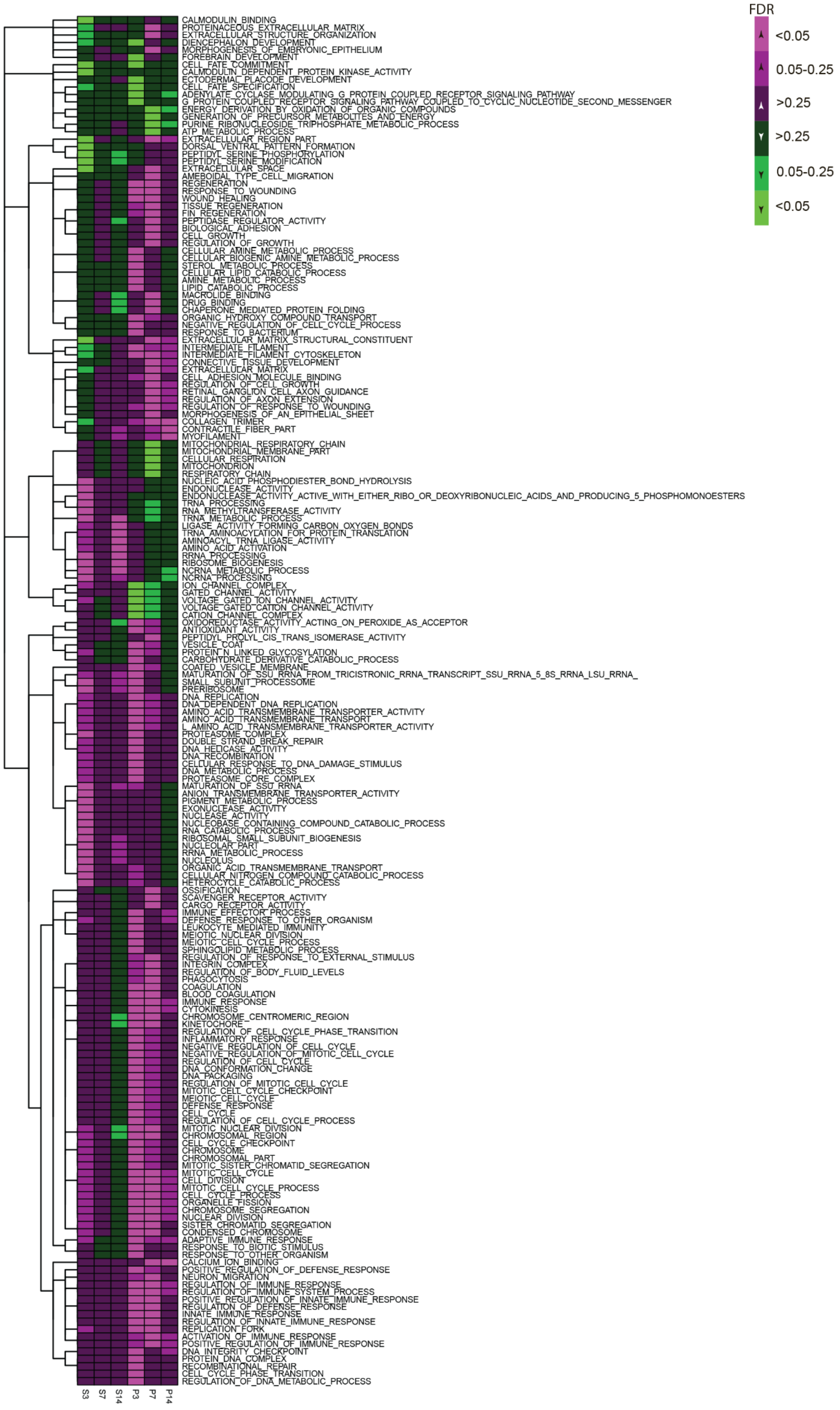


**Figure S1.** RNA sequencing GO term analysis, related to Figure 3. Heatmap of signed FDR values for all GO terms significantly different (FDR <0.05) between sham and at least one time point per population by pre-ranked GSEA. S, surface; P, Pachón; FDR, false discovery rate.

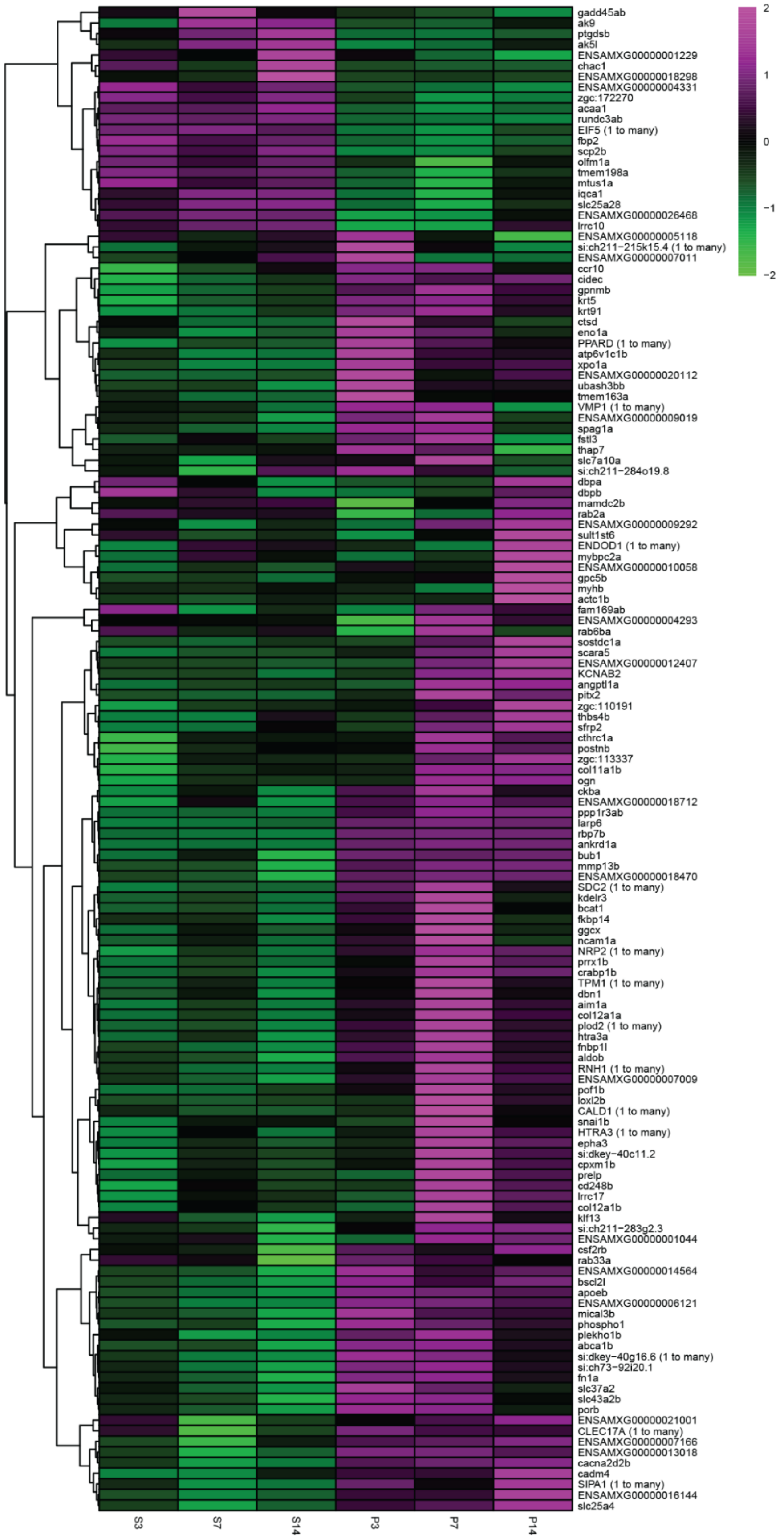


**Figure S2.** RNA sequencing analysis, related to Figure 3. Heatmap of row-normalised expression values for 141 genes with differential kinetics over the different time points clustered by Pearson correlation. Values are log2 fold change relative to sham. S, surface; P, Pachón.

| Gene | Forward primer sequence (5'- 3') | Reverse primer sequence (5'- 3') |
|---|---|---|
| ***caveolin*** | CAACGAGAGAGACCCACGAC | AGCTCATCCAACCTGACCAAC |
| ***ckba*** | CTTCCCAACGTGAGCAAACA | TGAGAACCCCAGACGGTCA |
| ***lrrc10*** | TCCCAACCTGAAAACCCTACAT | AGCTCATCCAACCTGACCAAC |
| ***snai1b*** | GCGCTAAAGATGCACATCCG | GTGAGGACAGGAGAACGGAC |
| ***sfrp2*** | AAGACGACAGTGAGATGGTGG | CGCCGTTCAGCTTGTATATGG |
| ***xpo1a*** | CAGGAGGCATTTTGTTCAGGTG | CGCCAATCATATAACCCACTGC |
| ***rpl13a*** **(housekeeping)** | TCTGGAGGACTGTAAGAGGTATGC | AGACGCACAATCTTGAGAGCAG |
| ***18s*** **(housekeeping)** | CGGAGGTTCGAAGACGATCA | GGAACCCAAAGACTCGTGGT |

**Table S1.** QPCR primer sequences, related to methods section 'Quantitative PCR'.
